# PTEN inhibits AMPK to control collective migration

**DOI:** 10.1038/s41467-022-31842-y

**Published:** 2022-08-11

**Authors:** Florent Peglion, Lavinia Capuana, Isabelle Perfettini, Laurent Boucontet, Ben Braithwaite, Emma Colucci-Guyon, Emie Quissac, Karin Forsberg-Nilsson, Flora Llense, Sandrine Etienne-Manneville

**Affiliations:** 1 Cell Polarity, Migration and Cancer Unit, Institut Pasteur, CNRS UMR3691, Université Paris Cité, Équipe Labellisée Ligue Contre le Cancer, F-75015 Paris, France; 2 Collège doctoral, Sorbonne Université, F-75005 Paris, France; 3 Macrophages and Development of Immunity Unit, Institut Pasteur, CNRS UMR3738, Paris, France; 4 Inserm U1127, CNRS UMR7225, Sorbonne Universités, UPMC University Paris 04 UMR S1127, Institut du Cerveau, ICM, Paris, France; 5 Department of Immunology, Genetics and Pathology and Science for Life Laboratory, Uppsala University, Uppsala, Sweden; 6 Present Address: Sorbonne Université, Institut de Biologie Paris-Seine (IBPS), CNRS UMR7622, 9 Quai St-Bernard, 75005 Paris, France

**Keywords:** Collective cell migration, Cell signalling, Cell invasion, Tumour-suppressor proteins

## Abstract

*Pten* is one of the most frequently mutated tumour suppressor gene in cancer. PTEN is generally altered in invasive cancers such as glioblastomas, but its function in collective cell migration and invasion is not fully characterised. Herein, we report that the loss of PTEN increases cell speed during collective migration of non-tumourous cells both in vitro and in vivo. We further show that loss of PTEN promotes LKB1-dependent phosphorylation and activation of the major metabolic regulator AMPK. In turn AMPK increases VASP phosphorylation, reduces VASP localisation at cell-cell junctions and decreases the interjunctional transverse actin arcs at the leading front, provoking a weakening of cell-cell contacts and increasing migration speed. Targeting AMPK activity not only slows down PTEN-depleted cells, it also limits PTEN-null glioblastoma cell invasion, opening new opportunities to treat glioblastoma lethal invasiveness.

## Abstract (web summary)

Pten is a tumour suppressor gene that is associated with highly invasive cancers such as glioblastoma. Here the authors show that PTEN loss results in increased migratory behaviour, which can be countered by targeting AMPK activity.

## Introduction

At the heart of a myriad of cellular processes, *Pten* (Phosphatase and TENsin homolog) is one of the most altered tumour suppressors in human cancer^1,2^. This holds particularly true in glioblastoma (GBM), the most malignant and frequent brain tumour, where *Pten* alteration is observed in 41% of cases^3–6^. PTEN is a dual-specific protein and lipid phosphatase and both activities are essential during development^7–9^. By dephosphorylating phosphatidylinositol-3, 4, 5-triphosphate (PIP_3_) into phosphatidylinositol-4, 5-bisphosphate (PIP_2_), PTEN antagonises the pro-oncogenic PI3K-Akt signalling pathway^10,11^ that is key to coordinate cell proliferation, growth, survival and metabolism. PTEN ability to regulate PIP_3_/PIP_2_ levels at the plasma membrane enables it to control cell polarisation and directionality during the directed migration of single cells^12–14^. PTEN protein-phosphatase activity also plays a role in PTEN functions but molecular details remain scarce^15^. Cancer spreading often requires collective motility^16^. Loss of PTEN function is associated with increased collective migration of breast epithelial cells, tumour aggressiveness and angiogenesis^6,17,18^. However, how PTEN effectively controls collective migration and invasion is still unclear. Since PIP_3_ is a key determinant of the basolateral surface, reduced PTEN activity has been proposed to alter epithelial characteristics, causing cells to switch to an invasive, motile, mesenchymal phenotype^19^. PTEN rescue experiments in cancer cell lines highlighted the importance of lipid phosphatase-independent activities, in particular in GBM cells^20–22^. In NIH 3T3 cells and U87 GBM cells, PTEN overexpression was shown to decrease cell migration and invasion possibly by reducing tyrosine (Y) phosphorylation of focal adhesion kinase (FAK)^20,23^.

To determine how the loss of PTEN promotes collective cell migration and invasion we down-regulated endogenous PTEN both in primary glial cells in vitro and in endothelial cells in vivo. We demonstrate that, during collective migration, PTEN depletion increases the speed of migrating cells and unravel a lipid-phosphatase independent connection between PTEN and the bioenergetics master regulator AMPK. This connection controls actin remodelling and cell-cell junctions to maintain cohesion and keep collective glial cell migration and invasion in check.

## Results

### PTEN loss increases collective cell migration

**Fig. 1:**
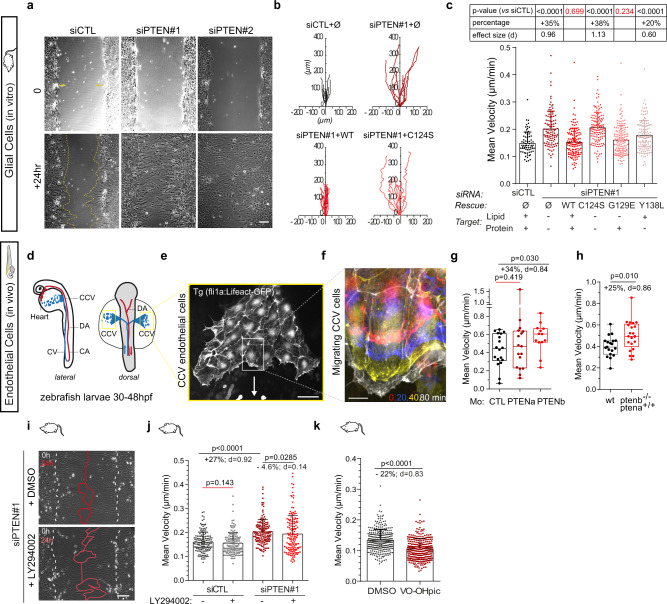
PTEN loss enhances collective cell migration independently of PI3K/AKT signalling. **a** Phase-contrast images of siCTL and PTEN-depleted (siPTEN#1,2) astrocytes migrating collectively in a wound-healing assay. Scale bar: 100 μm. **b** Diagrams showing 24 h-trajectories of 10 representative siCTL, and siPTEN#1 cells rescued with GFP alone (Ø), PTEN-WT(WT) and PTEN-C124S (C124S). **c** Mean astrocyte velocity. 69 siCTL cells, 107 siPTEN + Ø cells, 133 siPTEN+wt cells, 133 siPTEN+C124S cells, 145 siPTEN+G129E cells and 137 siPTEN + Y138L cells from three biologically independent experiments were examined. Statistics were drawn using two-tailed Mann–Whitney test. *p* values and effect sizes vs siCTL are given in the table above the graph. **d** Schemes representing lateral and dorsal view of a 24/30hpf zebrafish embryo with dorsal (DA) and caudal arteries (CA) in red and the caudal (CV) and common cardinal (CCV) veins. CCV endothelial cells (ECs) are delaminating from the midline, moving towards the heart (yellow box). **e** Representative fluorescent image of migrating ECs expressing Lifeact-eGFP. Scale bar: 50 μm. The micrograph is representative of at least 12 independent fish larvae. **f** Time-colored zoomed-in image of migrating ECs. Red is t = 0, blue is t = 20 min, yellow is t = 40 min and white is t = 180 min. Scale bar represents 10 µm. **g**, **h** Mean velocity of lifeact-eGFP expressing ECs in control zebrafish embryos (MoCTL), *ptena* morphant (MoPTENa) and *ptenb* morphant (MoPTENb) (**g**, *n* = 18, 17, 13 cells; *N* = 4 fish, two-tailed unpaired *t*-test) and in wild-type *pten* and in ptenb^−/−^ mutant zebrafish embryos (**h**, *n* = 19, 21 cells; *N* = 6, 5 fish; two-tailed unpaired *t*-test). **i** Phase-contrast images of siPTEN#1 cells treated with DMSO or the PI3K inhibitor LY294002. White dashed lines delineate the border of the wound at t = 0. Red lines delineate the border of the monolayer 24 h later. Scale bar 100 μm. **j**, **k** Mean velocity of siCTL and siPTEN cells treated with or without LY294002 (**j**, n = 198 cells, N = 3, two-tailed Mann–Whitney test) and with DMSO or VO-OHpic (**k**, n = 300, N = 3, two-tailed Mann–Whitney). Error bars represent Standard Deviation (SD). Boxes for box-plot graphs (**g**, **h**) extend from the 25th to 75th percentiles and the line in the middle is plotted at the median. Whiskers delineate all data points from minimum to maximum. Source data are provided as a Source Data file.

To address the effects of PTEN loss in collective migration we designed siRNAs against PTEN to decrease PTEN expression in rat astrocytes. siRNA efficiency was validated by observing a 64 and 79% decrease of total PTEN level and a sharp increase (>+120% and +80%) of PTEN-opposing PI3K pathway activity, highlighted by upregulation of pAKT level for siPTEN#1 and siPTEN#2, respectively (Supplementary Fig. 1a–d). Collective migration of glial cells was assessed using an in vitro wound-healing assay, which allows the quantitative assessment of cell speed and polarity^24^. Compared to control astrocytes (siCTL), PTEN-depleted cells (siPTEN#1/2) closed the artificial wound significantly faster (Fig. 1a, Supplementary Movie 1). The analysis of single cell tracks showed that PTEN-depleted wound-edge cells migrate longer distance than control cells (Fig. 1b). Quantification revealed PTEN loss strongly increases cell velocity (+32% for siPTEN#1 and +23% for siPTEN#2, Supplementary Fig. 1e) without strong defects in directionality and persistence of direction (Supplementary Fig. 1f, g). Expression of wild-type PTEN construct, but not of a dual phosphatase-dead mutant C124S (Supplementary Fig. 1i), significantly slows down the migration of siPTEN#1 astrocytes (Fig. 1b, c). Taken together these data reveal that deletion of PTEN is sufficient to increase cell speed during collective cell migration.

To assess whether PTEN’s role in collective migration was conserved in vivo, we looked at the endothelial cells (EC) forming the communal cardinal vein (CCV) during zebrafish (*Danio rerio*) early development using a transgenic line expressing Lifeact-eGFP under the fli1a blood-vessel specific promoter (Tg(fli1a:Lifeact-eGFP)^zf495Tg^)^25^. Starting around 20 h post-fertilisation, ECs delaminate from the midline and move collectively towards the heart, between the epidermis on top and the yolk sac syncytium layer underneath (Fig. 1d). ECs migrate as a single-sheet monolayer (Fig. 1e), in a cadherin-dependent fashion^26^, similarly to astrocyte monolayers closing an in vitro artificial wound, despite being much faster (Fig. 1f). A consequence of whole-genome duplication in teleosts, the zebrafish genome encodes two *pten* genes, *ptena* and *ptenb*, ubiquitously expressed and sharing partially redundant functions during early development^27,28^. We used morpholinos specific to *pten* orthologs^27^ and whose efficiency was validated here by the observation of whole body increased pAKT/AKT ratio (Supplementary Fig. 1h) and slight morphological defects (hooked tail^27^, Supplementary Fig. 1i) in both *ptena* and *ptenb* morphants at 2dpf. We observed that in *ptenb* morphants, but not in *ptena* morphants, ECs migrate faster than in control morphant fish (+34% increase, Fig. 1g). *Ptena* and *ptenb* share an identical phosphatase domain to human PTEN but differ in their membrane localisation C2 motif^27^, suggesting that difference in subcellular localisation may result in specific functions. However, because of a lack of anti-PTEN antibody suitable for immunofluorescence in zebrafish, we cannot exclude the possibility that *Ptena* is not expressed in ECs of the CCV at this stage. Finally, to rule out off-target physiological artifacts caused by ptenb morpholino^29^, we analysed CCV ECs velocity in ptenb^−/−^ mutant (*ptenb*^*hu1435*^)^28^ expressing lifeact GFP under the fli1a promoter and found that *ptenb* ECs migrate significantly faster (+25% increase, Fig. 1h). Altogether, our results show that PTEN limits cell speed during collective migration in different cell types and models.

### PTEN inhibits collective migration via its protein phosphatase activity

PTEN lipid phosphatase activity, which antagonises PI3K activity, is crucial to establish a front-rear gradient of lipids that sustain chemotaxis and directionality of amoeba and immune single cell migration^13^. We wondered whether it had a role in controlling glial collective cell migration or whether PTEN was acting via its protein phosphatase activity. By rescuing PTEN protein in PTEN-depleted astrocytes with either a lipid-phosphatase dead mutant (G129E) or a protein-phosphatase dead mutant (Y138L) (Supplementary Fig. 1j), we observed that only PTEN-G129E was able to rescue the mean cell velocity to siCTL level (non significant *p* value = 0.234 for G129E vs +20% difference and *p* < 0.0001 for Y138L, Fig. 1c). To further test the role of phospholipid signalling in the migration of PTEN-depleted cells, glial cells were treated with PI3K inhibitor LY294002. LY294002 treatment, whose efficiency is supported by a 80% drop in pAKT/AKT level compared to DMSO in control (Supplementary Fig. 1k, l) and siPTEN cells (Supplementary Fig. 1m), does not affect the speed of migration of PTEN-depleted cells (very weak effect size d = 0.14, and only −4.6% reduction, Fig. 1i, j). In addition, treatment with VO-OHpic, a potent inhibitor of PTEN lipid phosphatase activity that does not block protein phosphatase action of PTEN-like CX5R motif bearing phosphatase PTP1B^30,31^, strongly increases AKT phosphorylation (Supplementary Fig. 1k, l, Supplementary Fig 2b) but does not increase astrocyte velocity (Fig. 1k). Taken together, these data show that the increased cell velocity observed following PTEN depletion is independent of the PI3K/AKT pathway.

### PTEN loss alters interjunctional actin arcs in leader cells

**Fig. 2:**
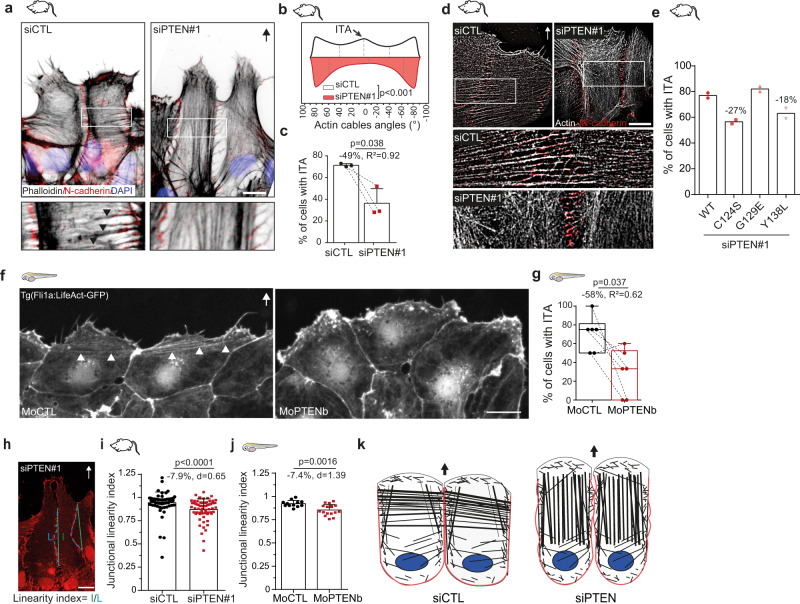
PTEN controls first-row cell-cell cohesion. **a**, **d** Normal (**a**) and super resolution (**d**) immunofluorescence images of actin filaments (phalloidin), cell-cell junctions (N-cadherin) in siCTL and siPTEN#1 migrating astrocytes. Black arrow: direction of migration. Boxed regions are zoomed in the panels below to highlight the presence of interjunctional transverse actin arcs (ITA) mostly in siCTL cells (black arrowheads). The micrographs in (**a**) are representative of the data shown in (**b**, **c**). The images in (**d**) are representative of six independent samples. **b** Angular distribution of actin filaments in front row siCTL and siPTEN#1 cells (*n* = 300). 0° is perpendicular to the direction of migration. Graph represents Kernel density estimates of probability density function. Dashed lines show quartiles. **c**, **e** Proportion of front row siCTL and siPTEN#1 cells with (**e**, *n* = 200) or without (**c**, *n* = 300) wt- and mutated-PTEN rescue, that are connected with ITA. **f** Fluorescence images of CCV ECs expressing LifeAct-GFP in CTL and *ptenb* morphant fish. White arrowheads point at ITA. **g** Percentage of front row CCV EC cells connected by ITA in MoCTL (*n* = 36 cells) and MoPTENb (*n* = 29 cells) fish larvae. Box extends from the 25th to 75th percentiles and the line in the middle of the box is plotted at the median. The whiskers delineate all data points from minimum to maximum. **h** Immunofluorescence image of N-cadherin in migrating astrocytes, where the linearity index of front row lateral cell-cell junction is defined. **i** Junctional linearity index in siCTL (*n* = 70) and siPTEN#1 (*n* = 65) cells, 8 h after migration. **j** Junctional linearity index in MoCTL (*n* = 12) and MoPTENb (*n* = 16) front row cells, 100 min after migration. **k** Schemes summarising PTEN loss phenotype in front row cells during collective cell migration. Scale bars: 10 µm. Number of independent experiments **=** 2 (**e**, **i**), =3 (**b**, **c**, **j**). Statistical tests: Kolmogorov-Smirnov (**b**), two-tailed paired *t*-test (**c**, **g**), two-tailed Mann–Whitney (**i**), two-tailed unpaired Student *t* test (**j**). *p* values, along with effect size coefficients are given on the graphs. Error bars represent SD. Source data are provided as a Source Data file.

Collective cell migration relies on the synchronisation of pathways permitting cytoskeleton remodelling and the maintenance of intercellular adhesion^32–34^. To unveil how PTEN loss leads to an increase in cell migration velocity, we investigated the impact of PTEN depletion on the actin cytoskeleton and the cell-cell junctions. In the front row of migrating astrocytes, F-actin form both longitudinal fibres anchored at the leading edge focal adhesions, and Interjunctional Transverse Arcs (ITA) that are oriented perpendicularly to the direction of migration and connect neighbouring cells through adherens junctions (AJ)^35^ (Fig. 2a, d). Interestingly, PTEN depletion leads to a loss of ITA (Fig. 2a–d) while actin cables parallel to the direction of movement become more pronounced as shown by the changes in the distribution of actin cables orientation within the cell (Fig. 2b). In vivo, front row of migrating ECs from control zebrafish morphants expressing LifeAct-GFP commonly show similar ITA (Fig. 2f and ref. ^36^). Depletion of PTENb decreased the number of cells connected by ITA (Fig. 2f, g). We then investigated if this phenotype was caused by the alteration of the lipid or protein phosphatase function of PTEN. Rescue experiments with PTEN mutants revealed that unlike the lipid-phosphatase activity, whose reactivation in siPTEN + PTEN-Y138L leads to the presence of ITA in only 63% of cells, compared to 77% in PTEN-wt rescued cells, the protein-phosphatase function of PTEN was sufficient to restore ITA in 82% of the +PTEN G129E cells (Fig. 2e). In parallel with the perturbation of ITA, PTEN depletion also decreased cell-cell junction’s linearity in both glial cells in vitro and ECs in vivo (Fig. 2h–j) and altered the formation of new junctions between leaders cells at the front (Supplementary Fig. 2b, c) which was shown to be linked to altered AJ recycling^35^. No alteration in AJ proteins level was observed in PTEN depleted glial cells (Supplementary Fig. 2a). Altogether, these data show PTEN regulates actin organization at cell-cell contacts via its protein phosphatase function and thus controls AJ recycling to support tighter connection between leader cells during collective migration (Fig. 2k).

### PTEN inhibits AMPK phosphorylation and activity

**Fig. 3:**
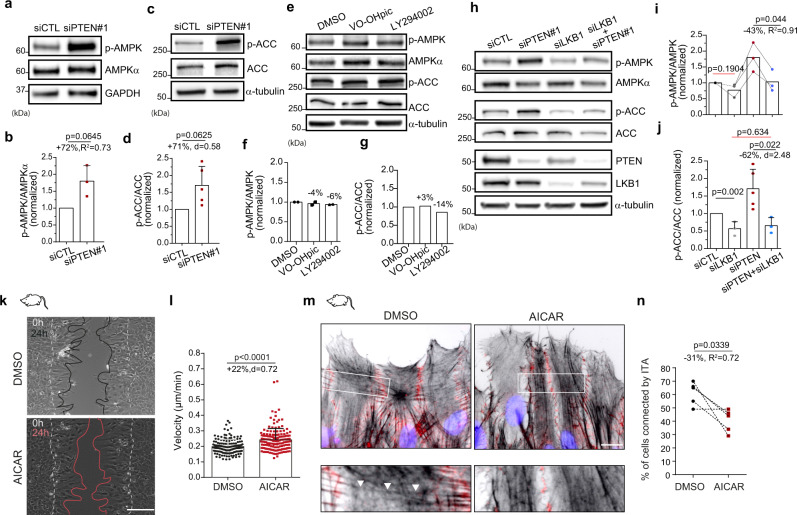
PTEN inhibits AMPK activity to control collective cell migration. **a**, **c**, **e** Western blot analysis of phosphorylated AMPK (T172), total AMPK, phosphorylated ACC (S79), ACC and GAPDH in siCTL and siPTEN#1 (**a**, **c**) and DMSO, VO-OHpic, LY294002-treated (**e**) astrocytes lysates. **b**, **f** Normalised ratio (over siCTL or DMSO) of T172 phosphorylation/total AMPK in siPTEN#1 (**b**, *N* = 3) and VO-OHpic- or LY294002-treated cells (**f**, *N* = 2). Two-sided paired *t*-test were realised on raw data to generate *p* values. **d**, **g** Normalised ratio (over siCTL or DMSO) of S79 phosphorylation/total ACC in siPTEN#1 (**d**, *N* = 5, Wilcoxon test) and VO-OHpic- or LY294002-treated cells (**g**, *N* = 1). **h** Representative western blot analysis of pAMPK (T172), total AMPK, ACC, pACC (S79), PTEN, LKB1 and α-tubulin in siCTL, siPTEN#1, si LKB1 and siPTEN#1 + siLKB1 astrocytes lysates. The analysis was repeated three times and analysed in (**i**, **j**). **i** Normalised ratio (over siCTL) of p-AMPK/AMPK (*N* = 3, two-tailed paired *t*-test on raw data). **j** Normalised ratio (over siCTL) of p-ACC/ACC (*N* = 3 for siLKB1 and siPTEN + siLKB1, *N* = 5 for siCTL and siPTEN, two-tailed unpaired *t*-test on raw data). Note that LKB1 depletion rescues basal AMPK activity in siPTEN#1 cells. **k** Representative phase-contrast images of DMSO and AICAR-treated astrocytes migrating in a wound-healing assay. White dashed lines delineate the border of the wound at t = 0. Black/Red lines delineate the border of the monolayer 24 h later. Scale bar: 100 µm. **l** Mean velocity of DMSO (*n* = 149) and AICAR-treated cells (*n* = 157). Data were acquired from three biologically independent experiments and analysed statistically using two-tailed Mann–Whitney test. **m** Immunofluorescence images of actin filaments (Phalloidin, black), cell-cell junctions (N-cadherin, red) and nucleus (DAPI, blue) in DMSO and AICAR-treated migrating astrocytes. Boxed regions are zoomed in the panels below to highlight the presence of ITA (white arrowheads) mostly in DMSO cells. Scale bar: 10 µm. **n** Proportion of front row DMSO and AICAR-treated cells connected by ITA. 300 cells over five biologically independent experiments were examined. Stasticial test: two-tailed paired *t*-test. Error bars represent SD. Full scan images of the blots and source data are provided as a Source Data file.

To understand the molecular mechanisms responsible for the role of PTEN protein phosphatase activity in the control of actin organisation and collective cell migration, we ran a small protein phosphorylation screen assay. We compared the ratio of protein phosphorylation in siPTEN#1 astrocytes cell vs siCTL with the ratio found in VO-OHpic vs DMSO-treated cells, to identify targets of PTEN potentially involved in the control of collective migration. Out of ~40 proteins of different signalling pathways, AMPKα (T172/T183) phosphorylation was increased by 17% in siPTEN#1 but not in VO-OH treated cells, a difference similar to what is seen for PTEN known target FAK (Y397) (+10%)^20,23^ (Supplementary Fig. 2a, b). Western blot analysis using a different set of AMPKα (T172) phospho-antibody, along with total AMPKα measurement, confirmed a strong increase in phosphorylation of AMPKα T172 in siPTEN#1 depleted cells (+72%, Fig. 3a, b) and in a second set of PTEN depleted cells, siPTEN#2 (+57%, Supplementary Fig. 3c, d). Since T172 phosphorylation is known to activate AMPK enzymatic activity^37^, we analysed the S79 phosphorylation of the biosynthetic kinase acetylCoA carboxylase (ACC), a classic substrate of AMPK^38^. PTEN loss strongly increased ACC phosphorylation, indicating an increased activity of AMPK (Fig. 3c, d, Supplementary Fig. 3e, f). In contrast, cell treatment with LY294002 and VO-OHpic did not affect AMPKα phosphorylation (Fig. 3e, f) nor its activity (Fig. 3e, g).These data reveal a causal link between PTEN loss and the activation of AMPK, a major guardian of cellular energy levels^39^; and thus more globally a functional link between PTEN and an energy production control hub.

The serine/threonine Liver Kinase B1 (LKB1) genetically and physically interact with PTEN (Supplementary Fig. 3g)^40,41^. Interestingly, LKB1 is one of the main kinases phosphorylating AMPKα on its T172 residue^42^. Decreasing LKB1 expression by siRNA in PTEN-depleted astrocytes restored AMPKα and ACC phosphorylation to control levels (Fig. 3h–j), showing that LKB1 is involved in the increased AMPK activity induced by PTEN depletion. Of note, LKB1 depletion lowers total PTEN protein level^43^ without increasing AMPK and ACC phosphorylation (Fig. 3h), confirming our conclusion that activation of AMPK in PTEN-depleted cells relies on LKB1. Taken together these data indicate LKB1 acts downstream of PTEN and is responsible for AMPK activation following PTEN loss.

To determine the importance of AMPK overactivation in directing the phenotype of migrating PTEN-depleted cells, we looked at the effect of AMPK pharmaceutical stimulation on glial cells migrating in a wound-healing assay. Cell treatment with AMPK activator 5-AminoImidazole-4-CArboxamide Ribonucleotide (AICAR), at low dose (40 µM), increases wound-healing closure and cell velocity during collective migration (+22%, Fig. 3k, l). In these conditions, we also observed a strong decrease in the percentage of cells connected by ITA (Fig. 3m, n), reminiscent of the reorganisation of actin cytoskeleton observed in PTEN-depleted cells.

### PTEN-induced AMPK activation delocalises VASP from AJs

**Fig. 4:**
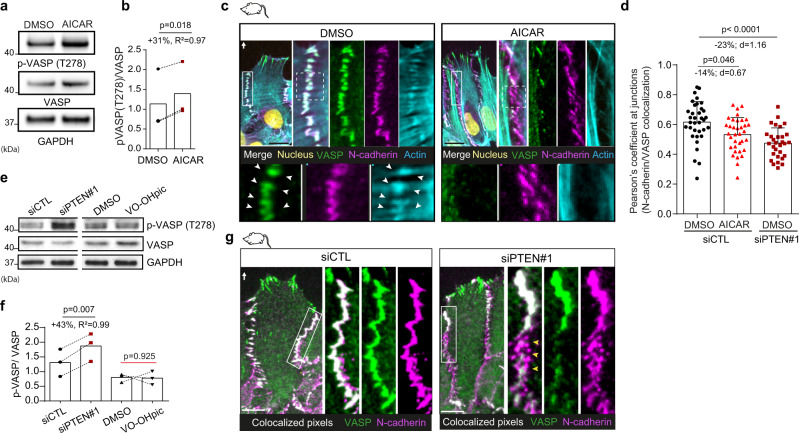
AMPK activation controls VASP phosphorylation and localisation at cell-cell junction. **a**, **e** Western blot analysis of phosphorylated VASP (T278), total VASP and GAPDH in DMSO- and AICAR-treated astrocytes (**a**) and siCTL, siPTEN#1, DMSO- and VO-OHpic-treated cells (**e**). **b**, **f** Ratio of pVASP/VASP in DMSO- vs AICAR-treated cells (**b**, +31%, *N* = 3, two-tailed paired *t*-test), in siCTL vs siPTEN#1 cells (**f**,+43%, *N* = 3, two-tailed paired *t*-test) and in DMSO- vs VO-OHpic-treated cells (**f**, *N* = 3, two-tailed paired *t*-test). **c** Immunofluorescence images of VASP (green), N-cadherin (magenta), F-actin (cyan) and DAPA (yellow) in DMSO- and AICAR-treated cells. White rectangles area are zoomed in the top panel. Dotted-line squares in the zoomed area are further zoomed in the bottom panels. Cell-cell junctions enriched in VASP associate with ITA anchoring (white arrowheads). Note that the absence of VASP at cell-cell junctions in AICAR-treated cells is associated with the absence of ITA. **d** Pearson’s coefficient of colocalized junctional N-cadherin with VASP in DMSO- and AICAR-treated siCTL cells (*n* = 35 cells analysed from three biologically independent experiments) and in DMSO-treated siPTEN cells (*n* = 30 cells analysed from three biologically independent experiments). Two-tailed unpaired *t*-test was used to derive *p* values. The drop in Pearson’s coefficient highlights the loss of VASP colocalisation with junctional N-cadherin in AICAR-treated and PTEN depleted cells. **g** Immunofluorescence images of VASP (green), N-cadherin (magenta) and colocalized pixels between N-cadherin and VASP (white) in siCTL and siPTEN#1 cells. White rectangles area are zoomed-in. Scale bars: 10 µm. Note the disappearance of colocalized pixels in large part of cell-cell junctions in siPTEN#1 cells (yellow arrowheads). Error bars represent SD. Full scan images of the blots and source data are provided as a Source Data file.

In search for downstream target of AMPK that could control actin cables and cell-cell junction dynamics, we focused on actin-binding proteins that are present at cell-cell junctions and can be phosphorylated by AMPK. The Vasodilator-Stimulated Phosphoprotein (VASP) met these criteria. VASP phosphorylation by AMPK occurs on T278 residue *in cellulo* and has been shown to impair actin stress fibres formation in EC^44^. AMPK activation following AICAR treatment increased VASP T278 phosphorylation in astrocytes (Fig. 4a, b). In migrating cells, VASP localised both at cell-cell junctions together with actin and N-cadherin (Fig. 4c, arrowheads in zoomed-in boxes) and at cell-ECM adhesion sites with paxillin and actin (Supplementary Fig. 4a, b). Increasing AMPK activity led to VASP delocalisation from N-cadherin-mediated AJs (Fig. 4c), as quantified by a significant drop in the fraction of N-cadherin overlapping VASP at lateral cell-cell contacts (Fig. 4d). In addition, we noticed that VASP presence at cell-cell junctions correlated with the presence of ITA (Fig. 4c, Supplementary Fig. 4c, yellow arrowheads). Interestingly, in AICAR-treated cells, patches of N-cadherin clusters lacking VASP were systematically devoid of ITA (Fig. 4c, zoomed-in white boxes).

We then tested whether PTEN depletion, which increases AMPK activity, could affect VASP in a similar way. PTEN loss significantly increased VASP (T278) phosphorylation, contrary to VO-OH-dependent lipid phosphatase inhibition (Fig. 4e, f) and altered VASP localisation at AJs (Fig. 4g), but not at focal adhesions (Supplementary Fig. 4b).We indeed observed a significant 23% drop of the Pearson’s coefficient assessing the colocalization between N-cadherin and VASP in siPTEN#1 cells compared to siCTL (Fig. 4d). Moreover, similarly to AICAR-treated cells, the absence of VASP at cell-cell junctions in siPTEN#1 cells was systematically associated with the absence of ITA at this specific location (Supplementary Figure 4d, yellow asterisks). Taken together these data reveal that AMPK activation mediates and phenocopies PTEN deletion during collective sheet-like migration. AMPK activation in PTEN-depleted cells increases VASP phosphorylation and triggers its relocalization away from cell-cell junctions, which is associated with destabilisation of ITA coupling neighbouring leader cells.

### AMPK inhibition reduces migration and invasion of PTEN-depleted cells

**Fig. 5:**
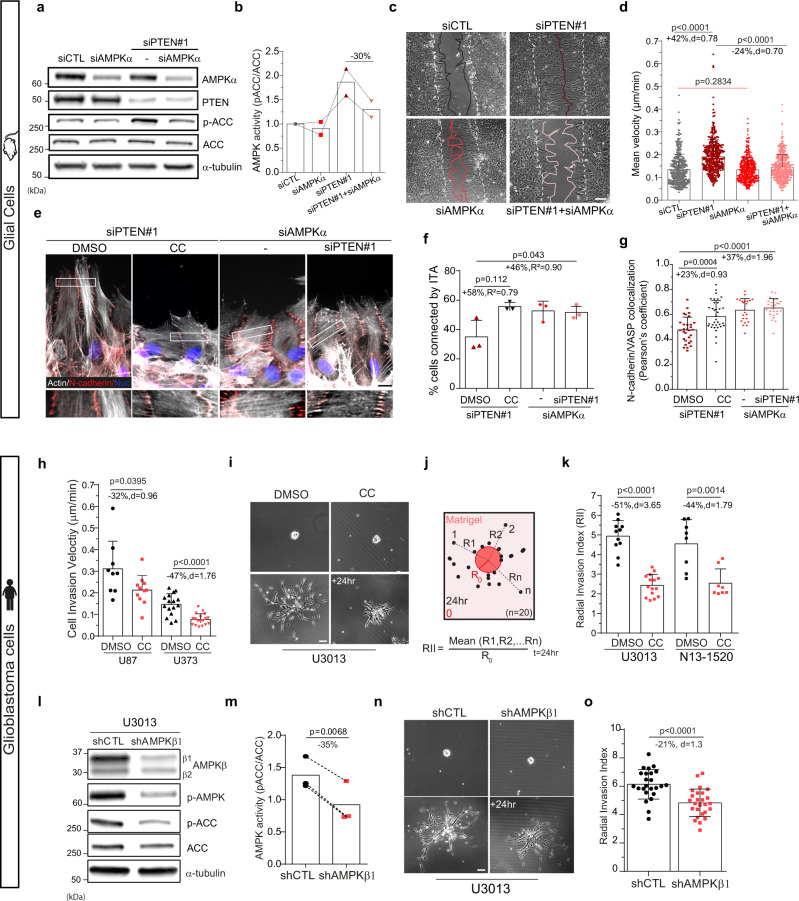
AMPK inhibition reduces collective cell migration and invasion efficiency by restoring leader cell cohesion. **a** Western blot analysis of siCTL, siPTEN#1, siAMPKα and siPTEN#1+siAMPKα astrocytes lysates. **b** Normalised ratio of pACC/ACC showing efficiency at reducing AMPK activity in double siPTEN+siAMPKα transfected astrocytes (−30%). **c** Phase-contrast images of same astrocytes as (**a**) migrating in a wound-healing assay. White dashed and coloured lines delineate the border of the wound respectively at t = 0 and t = 24 h. **d** Mean velocity of astrocytes shown in (**c**) during 24 h migration (siCTL: *n* = 374, siPTEN#1: *n* = 366, siAMPKa: *n* = 429, siPTEN#1+siAMPKa: *n* = 460 cells). **e** Immunofluorescence images of actin filaments (phalloidin, grey) and N-cadherin (red) in migrating astrocytes. **f** Proportion of leader cells connected by ITA in the same cells shown in (**e**). (siPTEN#1 + dmso: *n* = 481 cells, siPTEN#1 + CC: 456, siAMPKα: 392, siPTEN#1+siAMPKα: 508). Note that despite *p* value = 0.112, the effect size is important between DMSO- and CC-treated siPTEN#1 cells (+58%, *R*² = 0.79). **g** Pearson’s coefficient of N-cadherin/VASP colocalisation at lateral cell-cell junctions in cells shown in (**e**). (siPTEN#1+dmso: *n* = 30 cells, siPTEN#1+CC: 35, siAMPKα: 23, siPTEN#1+siAMPKα: 26). **h** Mean cell invasion velocity in a Matrigel^®^ spheroid assay of U87 and U373 cells treated with CC (U87/U373: DMSO, *n* = 108/184 cells from 9/16 spheroids; CC, *n* = 136/162 cells from 11/18 spheroids). **i** Phase-contrast images of DMSO- and CC-treated U3013 GBM cells just after being included in Matrigel^®^ and 24 h after (**j**). Scheme explaining calculation of the radial invasion index (RII). **k** RII of U3013 and N13-1520 cells treated with DMSO (*n* = 11 and 8 spheroids) or CC (*n* = 15 and 8 spheroids). **l** Western blot analysis of shCTL and shAMPKβ_1_ U3013 cell lysates. **m** pACC/ACC ratios showing −35% AMPK activity in shAMPKβ_1_ cells compared to shCTL. **n** Phase-contrast images of shCTL and shAMPKβ_1_ U3013 spheroids in Matrigel^®^ at t = 0 and 24 h after and their RII (**o**), showing a 21% decrease in infiltration efficiency when AMPKβ_1_ is depleted (*n* = 24/26 spheroids). Scale bars: 100 µm, except in (**e**), 10 µm. Number of biologically independent experiments **=** 2 (**b**), =3 (**d**, **f**–**h**, **k**, **m**, **o**). Statistical tests: two-tailed unpaired *t*-test (**d**, **g**, **h**, **k**, **o**), two-tailed paired *t*-test (**m**). Error bars represent SD. Full scan images of the blots and source data are provided as a Source Data file.

Finally, we asked whether AMPK inhibition could inhibit collective migration of PTEN depleted cells. To test this hypothesis, we used siRNA against AMPKα to decrease AMPK expression level and activity in PTEN-depleted cells (Fig. 5a, b; Supplementary Fig. 5a). In control primary astrocytes, AMPKα depletion did not affect significantly migration nor did it reduce ACC phosphorylation, which suggests that in these cells AMPK basal activity is low (Fig. 5a, b). In contrast, AMPKα depletion strongly reduced PTEN-depleted cells’ ability to close the wound (Fig. 5c, Supplementary Movie 2). Cell tracking measurements showed that AMPKα depletion reduces the migration speed of PTEN-depleted cells by 24%, which corresponds to a 43% decrease of the increase caused by PTEN loss (Fig. 5d). Similar results were obtained when the regulatory subunit AMPKβ was depleted in siPTEN#2 astrocytes (Supplementary 5b, c). In addition, inhibiting AMPK both with pharmacological inhibitor compound C (CC) or AMPKα depletion in PTEN-depleted cells rescued the formation of ITA (Fig. 5e, f) and the colocalization between junctional N-cadherin and VASP (Fig. 5g). These data show AMPK alteration is sufficient to rescue ITA-based leader cells connectivity and slow down PTEN-depleted collective cell migration.

The clear inhibitory effect of AMPK inhibition on the migration of PTEN-depleted cells led us to investigate whether inhibition of AMPK could reduce the invasion of PTEN-null cancer cells. Glioblastoma (GBM) are the most common and the most aggressive malignant brain tumours, thought to arise from glial cells at different stages of their differentiation status^45^. Highly invasive, GBM cells infiltrate the brain collectively in a connected network of cells^46^ or as diversely cohesive groups or chains of cells along the blood vessels and the myelinated nerve fibres^47–49^. We used PTEN null commercial GBM cell lines U87 and U373^50^, grown as spheroids and embedded in Matrigel. Inhibiting AMPK with CC treatment slowed down invasion speed significantly for both cell lines even though the inhibition appeared more pronounced in U373 (Fig. 5h). We then tested the impact of AMPK inhibition in primary patient-derived GBM cells devoid of PTEN (Supplementary Fig. 5d). AMPK inhibition strongly blocked the radial gel invasion of U3013 and N13-1520 cells (Fig. 5i–k, Supplementary Movie 3). To rule out potential off target effects of CC treatment and confirm the specific role of AMPK in the blocking of the invasion, we established a stable AMPKβ-depleted U3013 cell population (shAMPKβ1, Fig. 5l, Supplementary Fig. 5e). Although AMPK activity was only reduced by 35% (Fig. 5m), the invasive capacity of shAMPKβ1 U3013 cells was significantly inhibited (Fig. 5n, o). Together these data show that AMPK is a major actor in controlling migration and invasion in PTEN-depleted cells and suggest that AMPK inactivation may be sufficient to reduce PTEN-null GBM invasion.

## Discussion

In search of oncogenic events that could foster cell motility, we found that the loss of tumour suppressor PTEN alone is sufficient to enhance the collective migration of glial cells in vitro and EC in vivo. This effect is independent of PI3K/AKT signalling, but requires LKB1-dependent activation of AMPK, a master regulator of metabolism.

Whether collectively migrating PTEN-depleted cells require PI3K/AKT activation to increase their velocity seems to depend both on cell types and on the nature of the migratory stimuli. Contrary to what we observed in PTEN-depleted glial cells, and other on PTEN^+/−^ mouse astrocytes^51^, PTEN^−/−^ fibroblasts rely on PI3K/AKT-dependent Rac1 and Cdc42 activation to promote collective motility, in a similar wound-healing assay^12^. In single cell migration, PTEN loss results in PI3K/AKT activation and subsequent Rac1 stimulation in mouse embryonic fibroblasts and neutrophils^52–54^. The results found in astrocytes may be explained by the presence in these cells of alternative ways of activating Rac1, independent of PI3K/AKT. Alternatively, because cell-cell connective interactions vary between cell types during wound-healing assays, the differences between fibroblasts and astrocytes may rely on front row organisation of the monolayer during wound-healing closure.

Front row CCV EC and rat astrocytes form tight connections through interjunctional transverse actin cables (ITA). We found that front row PTEN-depleted cells lose ITAs and form less linear junctions possibly due to the decreased intercellular tension or to altered AJ dynamics. Cadherin-mediated interactions and actin network organization have been shown to be important for the directionality of CCV endothelial cell migration^26,36^. Here, the weaker connection between leader cells of PTEN-depleted embryo does not cause any alteration of global directionality, presumably due to maintenance of enough cell-cell junction’s integrity. However we observed a strong increase in cell velocity, which is in agreement with previous findings showing alteration of ITA affects adherens junction dynamics, which progressively leads to increased cell velocity^35^.

Mechanistically, we show here that during collective cell migration, PTEN alteration leads to LKB1-dependent activation of AMPK. LKB1 recruits AMPK to E-cadherin rich cell-cell contacts^55,56^, suggesting here that AMPK activation is spatially restricted at adherens junction in PTEN-depleted cells. VASP is present at AJ in migrating leader cells. Upon PTEN loss, we report that AMPK activation increases VASP phosphorylation on T278, and delocalises it from the adherens junction. In turn, increased T278-phophorylated VASP cytoplasmic accumulation near the adherens junction may alter F-actin elongation^44,57^, perturbing the formation of the interjunctional actin arcs. Alternatively, AMPK has been shown to regulate actomyosin contractility and junction maturation^55,58^. Increased contractile forces following AMPK activation may alter local balance of forces at N-cadherin-rich ITA anchoring points and thus participate to their detachment. The fact that AMPK inhibition by the CC or by siRNA-mediated depletion, totally rescues the formation of ITAs (Fig. 5e, f) indicates that AMPK is the major effector of PTEN that control actin organization at AJs. However, it is possible other downstream target of PTEN protein phosphatase activity, such as FAK, cofilin or Abi1^20,59,60^, may contribute, independently of AMPK, to the change in actin organization in migrating PTEN-depleted cells.

Additional pathways controlled by AMPK activation may also contribute to the alteration of collective cell migration. Loss of AMPK was shown to increase surface adhesion and spreading^61^, suggesting increased AMPK activity might have the opposite effect, decreasing matrix attachment and thus promoting migration. Increased FAK (Y397) phosphorylation in PTEN depleted cells would further alter cell-ECM attachment.

PTEN is known to affect cellular bioenergetics and cell growth via its negative regulation of PI3K/AKT-dependent control of mammalian target of rapamycin signalling^62^. We unveil here, in glial cells, a PI3K-independent function of PTEN in metabolism control, via its upregulation of AMPK activity. AMPK-dependent metabolic pathways also affects cell migration velocity by regulating intracellular ATP:ADP ratio^63^. AMPK-dependent enhanced energy production at the leading edge of migrating cells also sustains cell motility machinery by controlling polarised trafficking of mitochondria at the front of the cells and lamellipodia turnover^64,65^ and promotes the lifetime of leader cells^66^.

Our study brings insights into how PTEN alteration could drive cancer progression. Because sheet-like migration is often seen in tumour invasive front in vivo, notably in skin and intestine tumours^67^, and in perivascular environment for some GBM cells^68^, we believe the uncovering of AMPK’s role in mediating the effect of PTEN loss offers a potential therapeutic route to tackle cancer cell invasion. While AMPK activity has long been seen as suppressing cancer progression by slowing down cellular growth and proliferation^69–71^, it was recently shown to be hyperactivated in GBM and promote its development by modifying cellular bioenergetics^72^. We show here that targeting AMPK also reduces GBM cell invasion, reinforcing the interest in developing AMPK inhibitors to treat GBM.

## Methods

### Zebrafish lines and husbandry

Zebrafish (*Danio rerio*) of the AB background (Wt, from the Zebrafish International Resource Center) IRC, Eugene, OR, USA), the *ptenb*^*hu1435*^ mutant line^28^ and the transgenic line Tg(fli1a:Lifeact-eGFP)^zf495Tg^ ^25^ were raised according to standard procedures with a 14 h light/10 h dark cycle as previously described^73^. Eggs obtained by natural spawning were bleached and raised at 28 °C in Volvic source water supplemented with 280 μg/L of methylene blue (Sigma Aldrich, Cat#: M4159). N-Phenylthiourea (PTU, Sigma Aldrich, Cat#: P7629) was added to raising medium (0.003% final) from 24 hpf onwards to prevent pigmentation and facilitate imaging. Animal experiments were conducted according to European Union guidelines for handling of laboratory animals (http://ec.europa.eu/environment/chemicals/lab_animals/home_en.htm). All protocols were approved by the Ethical Committee for Animal Experimentation of Institut Pasteur – CEEA 89 and the French Ministry of Research and Education (permit #01265.03). During injections or live imaging sessions, animals were anaesthetised with Tricaine (Ethyl 3-aminobenzoate methanesulfonate, Sigma-Aldrich, Cat#: A5040). At the end of the experimental procedures, they were euthanized by anaesthetic overdose.

### Mammalian cell culture

Primary astrocytes were obtained from E17 rat embryos as previously described^24^. Cells were grown in 1 g/L D-glucose DMEM + Pyruvate supplemented with 10% FBS (ThermoFischer Scientific, Waltham, MA), penicillin-streptomycin (100 U ml^−1^ and 100 μg ml^−1^, Gibco^™^) and Amphotericin B (2.5 µg/ml, Gibco^™^) at 5% CO_2_ and 37 °C. Medium was changed 1 day after transfection and 1 day before the experiments. Human Neural Stem Cells were obtained from ThermoFischer Scientific (Gibco^™^, ref#N7800100, H9-derived) and cultured as recommended using KnockOut^™^ DMEM⁄F-12 Basal Medium, StemPro™ Neural Supplement, human FGF-basic and human EGF (animal free, Peprotech) at 20 ng/mL. Commercial GBM cell lines U87 and U373 were grown in modified Eagle’s medium (MEM) supplemented with 10% FBS, penicillin-streptomycin and non-essential amino-acids (all from Gibco^™^). U3013 and N13-1520 primary human GBM cells were acquired respectively from The Human Glioblastoma Cell Culture Resource (Uppsala University, Sweden, www.hgcc.se)^74^ and GlioTex (Institut du Cerveau et de la Moelle Epinière, ICM, F-75013, Paris, France)^75^; both institutions having the necessary ethical agreements to collect GBM samples from informed patients. Once thawed, both cell types were plated on Geltrex^™^ (Gibco) pre-coated plates and grown using serum-free GBM complete medium consisting in Neurobasal-A medium mixed 1:1 with DMEM/F12 + GlutaMAX and complemented with human FGF-basic and human EGF (animal free, Peprotech) at 20 ng/mL, and B-27^®^ (Gibco). HEK-293T were grown using DMEM + GlutaMAX + 4.5 g/L D-glucose + Pyruvate (Gibco^™^) medium complemented with 10% FBS and penicillin-streptomycin. Cells were routinely tested for mycoplasma contamination using PCR detection kits.

### Gene depletion and transfection protocols

Astrocytes were transfected with siRNAs (1–5 nM) or plasmids (5 µg) using a Nucleofector machine (Lonza) and the appropriate Lonza glial cell nucleofector solution. Transfected cells were then plated on appropriate supports previously coated with poly-L-Ornythin (Sigma) and experiments were performed 4 days post-transfection, when optimal protein silencing or expression was observed. Sequences of siRNAs used here are: siCTL (luciferase): UAAGGCUAUGAAGAGAUAC; siPTEN#1:AGGACGGACUGGUGUAAUGUU; siPTEN#2:GUGGAAAAUGGAAGUCUUUGUGA, siLKB1:GCUCUUUGAGAACAUCGGG, siAMPKβ1/2:UUUGAAGUAUUUGAUGCUUUAA. siAMPKα1 consisted in the ON-TARGETplus SMARTpool against rat PRKAA1 (Dharmacon^™^,Ref#SO-2905147G). To generate stable primary human GBM cells devoid of AMPK (shAMPKβ1) and the shCTL control clone, GBM#U3013 cells were infected with lentiviral particles generated by transfecting HEK293 cells with pLKO.1-puro plasmids from the Mission shRNA library (Sigma-Aldrich). Briefly, lentiviral particles were added to the plated cells for 24 h, before cells were washed with GBM complete medium. 2 days later 3 µg/ml puromycin was added to select positively infected cells. Antibiotic selection was prolonged for several days until the separate uninfected plated cells, seeded at the same concentration, were all dead. Several shRNA sequences against AMPKβ1 from the Mission library were tested and the one inducing the maximal protein depletion was kept (Ref#TRCN0000004770). pLKO.1-puro non-target shRNA (Sigma-Aldrich) was used to produce the shCTL clone.

PTEN rescue experiments were realised using pHR-SIN-PTEN lentiviral constructs (WT, C124S, G129E and Y138L) deposited on Addgene by Todd Waldman lab (Addgene plasmids #30370, 30376, 30377, 30378, respectively). Lentivirus infection efficiency was assessed after each experiment by PTEN and pAKT/AKT level measurement. Briefly, lentiviral particles were added to siPTEN transfected astrocytes 24 h after transfection and left for 24 h before being washed out and replaced by culture medium. Cells are imaged 3 days after lentiviral infection. pEGFP-C1-LKB1 (LKB1-GFP) plasmid was kindky provided by M.Salmi.

### Lentivirus production

Lentiviral particles were generated by transient transfection of HEK 293 T cells. In brief, 7 × 10^6^ cells were transfected with a mixture of DNA/CaCl_2_ diluted 1:1 in 20 mmol.l^−1^ HEPES after 20 min of incubation at room temperature. DNA comprised a mix of 10 µg 2nd generation packaging plasmid psPAX2, 5 µg of viral envelope plasmid pMD2.G and 10 µg of plasmid of interest (pLKO.1-puro, pGK-GFP or pHR-SIN plasmids). Viral supernatant was harvested 36 and 48 h post-transfection and concentrated using ultracentrifugation (Beckman Coulter, Optima XPN-80) for 1.5 h at 19,000 *rpm* and 4 °C and then stored at −80 °C. Viral particles were then dropped directly in the targeted cells medium, at 1 in 5000 dilution.

### Zebrafish morpholino microinjections

Zebrafish embryos in E3 medium (5 mM NaCl, 0.17 mM KCL, 0.33 Mm CaCl_2_ and 0.33 mM MgSO_4_) were injected using a Picospritzer III microinjector (Parker Hannifin) and a mechanical micromanipulator (M-152; Narishige, Tokyo, Japan). Morpholinos were loaded in a pulled borosilicate glass filament-containing capillary (GC100F-15; Harvard Apparatus, Holliston). Typically 1 nL was injected in 1-cell-stage embryos, at room temperature. 2 ng of Ptenb and Ctl morpholinos and 1 ng of *Ptena* morpholinos were delivered per injection, as defects in development were too severe when 2 ng was used for *Ptena*. The sequences of the Morpholinos are: *Ptena*: CCTCGCTCACCCTTGACTGTGTATG; *Ptenb*: CTTTCGGACGGTCGGTCGTCTTTA. A Standard Control oligo from the company was used as control (Gene Tools,LLC, Philomath, OR, USA).

### Drugs treatments and chemicals

For glial cells, drugs were added 1 h before the start of the experiments. VO-OHpic (Tebu-bio, Ref#B-0351), CC also called Dorsomorphin (EMD, Millipore, Ref#171261), and LY294002 (Calbiochem^™^) were used at 10 µM. AICAR (Enzo, Ref#BML-EI330-0050) was used at a low dose of 40 µM.

### In vitro migration and invasion assays

For scratch-induced wound-healing migration assays, cells were seeded on poly-L-ornithine coated coverslips (for immunofluorescence), 35 mm-diameter glass-bottom MatTek^®^ culture dishes (for fluorescent videomicroscopy) or 12-well plates (for brightfield videomicroscopy), and grown to confluence. On the day of the experiment, the monolayer of cells is scratched with a blunt-ended microinjection needle, creating a 300/500 nm-wide wound that is closed up by cell’s collective migration. For immunofluorescence staining, cells are allowed to migrate for 8 h before fixation. To assess collective cell migration kinetics, 24 h movies with a 15 min time-lapse interval are recorded using brightfield videomicroscopy performed by a Nikon Eclipse Ti2 epifluorescence inverted microscope equipped with a pco.edge 3.1 sCMOS camera (PCO, Germany) in a humidified, CO_2_-controlled heating chamber (5% CO_2_ and 37 °C, Okolab, Italy). All images were acquired with a dry 10 × 0.45 NA objective by the MetaMorph^®^ Microscopy Automation and Image Analysis Software (Molecular Devices, CA, USA).

Radial 3D invasion assays were performed by embedding GBM spheroids into a 50% Matrigel^®^ solution (Corning^®^, Merck) (1:1 Matrigel^®^ diluted in spheroids + GBM medium). Their efficiency at disseminating within the gel is analysed for 24 h, by acquiring brightfield images every 15 min. Glioblastoma (GBM) spheroids are generated by growing GBM cells in non-adherent flasks with the same GBM complete medium for a minimum of 2–3 days, until the spheroids reach ~100/200 µm in diameter.

### In vivo migration

For in vivo imaging, five to ten 30–48 h post-fertilisation zebrafish embryos were manually dechorionated with forceps, anaesthetised with 112 µg/ml Tricaine, immobilised in 1% low-melting-point agarose supplemented with 1xTricaine, in the centre of a 35 mm glass-bottomed dishes (MatTek Life Sciences, MA, USA), and then covered with ∼2 ml Volvic water containing 0.2× Tricaine. Fluorescence imaging of the Tg(fli1a:Lifeact-gfp) strain was performed using a spinning-disk confocal microscope (UltraVIEW vox, PerkinElmer) composed of a Zeiss AxioObserver Z1 stand equipped with a spinning-disk head Yokagawa CSUX1, two EMCCD cameras (Hamamatsu, Japan) and a humidified, C0_2_ controlled, heating chamber. We used either a 63× or a 40× oil-immersion objective to collect 0.5 µm z-stack images every 2 min for 1–2 h, using the Volocity^®^ software. We did not observe any difference in the proportion of CCV EC leader cells connected by ITA at 30 and 48 hpf.

### Migration and invasion kinetics measurement

Manual Tracking plugin (FIJI, ImageJ^76^) was used to analyse collective cell migration and 3D Matrigel^®^ invasion characteristics by tracking the nucleus of non-dividing leader cells located at the wound/spheroid edge. Velocity, directionality and persistence of direction were calculated following a previously published protocol^77^. Between 50 and 100 cells in randomly chosen part of the wound were analysed per repeat experiment. Radial invasion index was calculated based on t0 and t + 24 h images, as explained in the Fig. 5j. Briefly, the mean radius (i.e, the distance between the cell body to the centre of the spheroid) at 24 h of the 20 most spread cells was measured and normalised by the radius of the spheroid at t0.

### Immunofluorescence

Cells migrating for 8 h were fixed in cold methanol for 3 min at −20 °C or 4% PFA for 10 min at 37 °C, permeabilised for 10 min with 0.2% Triton and blocked with 3% BSA in PBS for 1 h at room temperature. Cells were then incubated for 1 h with primary antibodies diluted in PBS 1X, washed three times in PBS, and incubated another hour with secondary antibodies. Finally, coverslips were washed and mounted in ProLong Gold with DAPI (Thermo Fisher Scientific). The following primary antibodies were used: anti–α-tubulin (1/500, MCA77G YL1/2, rat monoclonal; Bio-Rad), anti-GAPDH (1/2000e, mouse, Chemicon International, MAB374), anti-Paxillin (1/200, mouse, BD transduction, #610051), anti–N-cadherin (1/1000, ab12221, rabbit polyclonal, lot GR139340-26, Abcam and sc-31030, 1/200 for immunofluorescence, goat polyclonal, clone K-20, lot B2014, Santa-Cruz), anti-αE-catenin (1/1000, Rabbit, Cell Signaling 3236S), anti-βcatenin (1/1000, Mouse, BD,#610154, lot:3137536), anti-p120ctn (1/1000, Mouse, BD, #610134, lot:71996), anti-P-Akt (1/1000e, Rabbit, Cell Signaling 4060S), anti-Akt (1/1000, rabbit, Cell Signaling 4685S), anti-AMPKα (1/1000, Rabbit, Cell Signaling 2532 S), anti-AMPKβ (1/1000, Rabbit, Cell Signaling 4150 S), p-AMPKα/T172 (1/1000, Rabbit, Cell Signaling 2585 S), ACC (1/1000e, Rabbit, Cell Signaling, 3662 S), p-ACC (1/1000, Rabbit, Cell Signaling 3661 S), LKB1 (1/1000, Rabbit, Cell Signaling, 3047),VASP (1/1000 for immunoblot and 1/200 for immunofluorescence, Rabbit, Cell Signaling 3132), P-VASP/T278 (1/1000, Rabbit, Sigma Aldrich, SAB4200521), GFP-HRP (1/5000, Abcam, Ab6663). Phalloidin-iFluor647 reagent (1/400, Abcam Ab176759) was used to label F-actin filaments. Secondary antibodies were Alexa Fluor 488 donkey anti-rabbit (711-545-152), Rhodamine (TRITC) donkey anti-rabbit (711-025-152), Rhodamine (TRITC) donkey anti-mouse (715-025-151), Alexa Fluor 647 donkey anti-rabbit (711-695-152), Alexa Fluor 647 donkey anti–goat (705-605-147), and Alexa Fluor 488 donkey anti–rat (712-545-153); from Jackson ImmunoResearch, all at a dilution of 1/10,000. Epifluorescence images were obtained on a microscope (DM6000; Leica Biosystems) equipped with 40×, 1.25 NA, and 63×, 1.4 NA, objective lenses and were recorded on a charge-coupled device camera using Leica Biosystems software. Super-resolution images (Fig. 2d) were acquired xith Zeiss LSM780 ELYRA with 63 × 1.4 NA or 100 × 1.46 NA objectives and recorded on an EMCCD Andor Ixon 887 1 K with Zen software.

### Electrophoresis and western blot

Glial cells are lysed with Laemmli buffer composed of 60 mM Tris pH6.8, 10% glycerol, 2% SDS and 50 mM DTT with the addition of either a 10x phosphatase cocktail inhibitor made of 200 mM Napp, 500 mM NaF, 10 mM sodium orthovanadate and 20 mM EDTA, or PhosSTOP^™^ (Sigma-Aldrich). Samples are then boiled for 5 min at 95 °C before loading on polyacrylamide gels. Transfer is performed at 0.1 A constant overnight or at 0.3 A for 2 h at 4 °C on nitrocellulose membranes. Finally, membranes are blocked with 5% milk or BSA for phosphorylated proteins in TBS + 0.2% Tween^®^20 (Sigma-Aldrich) for 1 h and incubated 1 h with primary antibody at room temperature. After being washed three times in TBST, they are incubated 1 h with HRP-conjugated secondary antibody. Bands are revealed with ECL chemiluminescence substrate (Pierce, Thermoscientific). Total and phosphorylated proteins were loaded on separate gels and housekeeping gene (GAPDH, beta-actin or alpha-tubulin) were revealed for each gels to compensate for potential loading differences. Zebrafish lysis to monitor morpholinos efficiency in vivo were done by dechorionating and deyolking 24/48hpf larvae in deyolking buffer (55 mM NaCl, 1.8 mM KCL, 1.25 mM NaHCO_3_ and 0.5 mM EDTA) on ice before adding Laemmli supplemented with DTT (0.1 M) and phoSTOP^™^ (Roche, Merck KGaA, Darmstadt, Germany), mechanically grinding the larvae using a disposable pellet mixer (VWR, #47747-370, Radnor, PA, US) and boiling the lysates for 5 min. Data analysis was performed using the Gels plugin (FIJI). Immunoblots are labelled with molecular weights markers given in kDa.

### Immunoprecipitation

Confluent 10 cmØ dishes of transfected HEK293 cells s were washed with cold 1×PBS and lysed with 1 ml of 1× IP buffer (500 mM Tris HCL pH 7.5, Triton 20%, 2 M NaCl) with the fresh addition of cOmplete^™^ protease inhibitor cocktail (Roche). Lysates were centrifuged at 13,000 *rpm* 2.30 min at 4 °C. Some supernatant mixed 1:1 with 2× Laemmli buffer was stored at −20 °C to serve as Input loading control. The rest of the supernatant was incubated for 2 h at 4 °C on the spinning wheel with Protein G beads (50 ul/dish) and 1 µg of homemade GFP-GST nanobodies collected from BL21 bacteria transfected with pGEX-GST-GFP, or GST beads only. Beads were then washed eight times with IP washing buffer (50 mM Tris HCL pH 7.5, 150 mM NaCl, 1 mM EDTA, 2.5 mM MgCl2) before adding Laemmli buffer and loading on precast gels (Invitrogen).

### Proteome profiler array

The human Phospho-kinase array kit (R&D Systems, Ref#ARY003B) was used to perform the small phosphoprotemics screen to unveil targets affected by PTEN depletion. Experiments and analysis were realised in accordance with the provider’s protocol.

### Immunofluorescence image analysis

*Angular distribution of actin filaments* in cells was measured as follow. After defining a reference orientation parallel to the wound, leader cells were segmented manually based on the N-cadherin staining and OrientationJ plugin was used to extract local orientations of F-actin filaments based on Phalloidin staining. Kolmogorov-Smirnov test on the non-normal distribution of data was performed to validate the differences between siCTL and siPTEN.

#### Interjunctional transverse actin arcs (ITAs)

Leader cells in the front row were scored manually as being connected by ITA if at least two actin arcs anchored at cell-cell junctions on both side of the cell could be seen.

#### VASP/N-cadherin colocalisation

VASP presence at cell-cell junctions was monitored by measuring its colocalisation with junctional N-cadherin. ROI was drawn around lateral cell-cell border from leading edge to roughly just in front of the nucleus. Then, the Pearson’s coefficient were measured on thresholded immunofluorescence images using JACOP (Just Another Colocalization Plugin) plugin in FIJI. One data point corresponds to the mean of the left/right junction.

*Lateral cell-cell junction linearity* was defined as the ratio between the length (straight line between the further at the rear to the most in front cell-cell contact) and the actual distance of the lateral intercellular contacts (cell-cell junction contour), based on the N-cadherin staining (Fig. 2h). Linearity index is 1 when the cell-cell junction is perfectly straight.

All data are presented as the mean ± standard deviation of at least three independent experiments, unless otherwise stated.

### Statistical analyses

Statistical analysis were obtained with two-tailed unpaired or paired Student’s *t* test depending on the type of experiment conducted, when data followed a Gaussian distribution (assessed by D’Agostino and Pearson normality test). When data failed this test even after cleaning data for outliers using the ROUT method or by transforming the raw data (using the logarithms), a Mann–Whittney non parametric analysis was performed. Statistical analysis for western blot data was done on the non-normalised ratio ([phosphorylated protein#1/housekeeping gene#1] / [total protein#2/housekeeping gene#2]) using paired *t*-test when three or more experiments were done. Quantification and statistical analysis were realised using GraphPad Prism 6 software. *p* values measurement from the appropriate statistical tests, a measure of effect size (Cohen’s coefficient, d, or the partial eta squared, *R*^2^,for paired-*t*-test) and the percentage difference between the two groups of interest appears on each graphs. Error bars on each graph represent standard deviation.

### Reporting summary

Further information on research design is available in the Nature Research Reporting Summary linked to this article.

## Supplementary information

Supplementary Information

Description of Additional Supplementary Files

Supplementary Movie 1

Supplementary Movie 2

Supplementary Movie 3

Reporting Summary

## Data Availability

All relevant data are available within the paper and the Supplementary materials. Source data are provided with this paper.

## Source data

Source Data
