# Supplementary material for: PTEN inhibits AMPK to control collective migration
Source: Nat Commun. 2022 Aug 11;13:4528. doi: 10.1038/s41467-022-31842-y (PMC9372137; doi:10.1038/s41467-022-31842-y)
Supplement: Supplementary file 1 — Supplementary Information [file 41467_2022_31842_MOESM1_ESM.pdf]

Supplementary Figure 1.

a. Western blot analysis of PTEN and α-tubulin in siCTL and siPTEN#1/#2 astrocytes lysates. Bands are from the same blot but not originally next to each other. Original blots can be found in Open Source Data file. **b**. PTEN protein expression level in siPTEN#1/#2 cells (normalized to siCTL, n=2/n=4 biological replicates). **c.** Western Blot analysis of phosphorylated-AKT (S473), AKT and GAPDH in siCTL and siPTEN#1/#2 astrocytes **d.** Phospho-AKT/AKT ratio level in siPTEN#1 and siPTEN#2 cells increases by 120% and 80% compared to siCTL cells (n=4 and n=2 biological replicates, two-tailed paired t-test). **e.** Mean velocity of siCTL and siPTEN#1,2 cells (n=199 cells from 3 biological replicates and n=80 and n=91cells, from 2 biological replicates, two-tailed Mann Whitney test). **f**. Directionality and **g.** Persistence indexes (see Material and Methods) in siCTL and siPTEN#1 astrocytes migrating in a wound-healing assay (n=150 and 147 cells from 3 biological replicates, two-tailed Mann-Whitney test). **h.** Representative western blot analysis of pAKT and AKT in *pten* morphants. **i.** Representative images of 2dpf zebrafish morphants morphology (n=3 biological replicates). White arrow points at hooked tail in MoPTENb. scale bar 0.5mm. **j.** Representative western blot analysis of PTEN, pAKT, AKT and GAPDH in siCTL and siPTEN#1 astrocytes infected with lentivirus expressing GFP (Ø), PTEN (WT), PTEN-C124S, PTEN-G129E and PTEN-Y138L. Note that the ectopic expression level of mutants without lipid-phosphatase function is sufficient to affect PI3K signalling (C124S and G129E) and mutant without only protein-phosphatase function has no impact on PI3K signalling (n=3 biological replicates). **k.** Western Blot analysis of PTEN, pAKT, AKT and GAPDH in astrocytes treated with DMSO, VO-OHpic and LY294002. **l.** Normalized pAKT/AKT ratio level in drug-treated astrocytes confirms efficiency of the drugs at inhibiting (LY294002, n=3 biological replicates) or increasing (VO-OHpic, n=5) PI3K signalling. **m.** Representative Western blot analysis of PTEN, pAKT, AKT and GAPDH in siCTL and siPTEN astrocytes treated with or without LY294002 (n=2 biological replicates). Error bars represent SD. Full scan images of the immunoblots and source data are provided as a Source Data file.

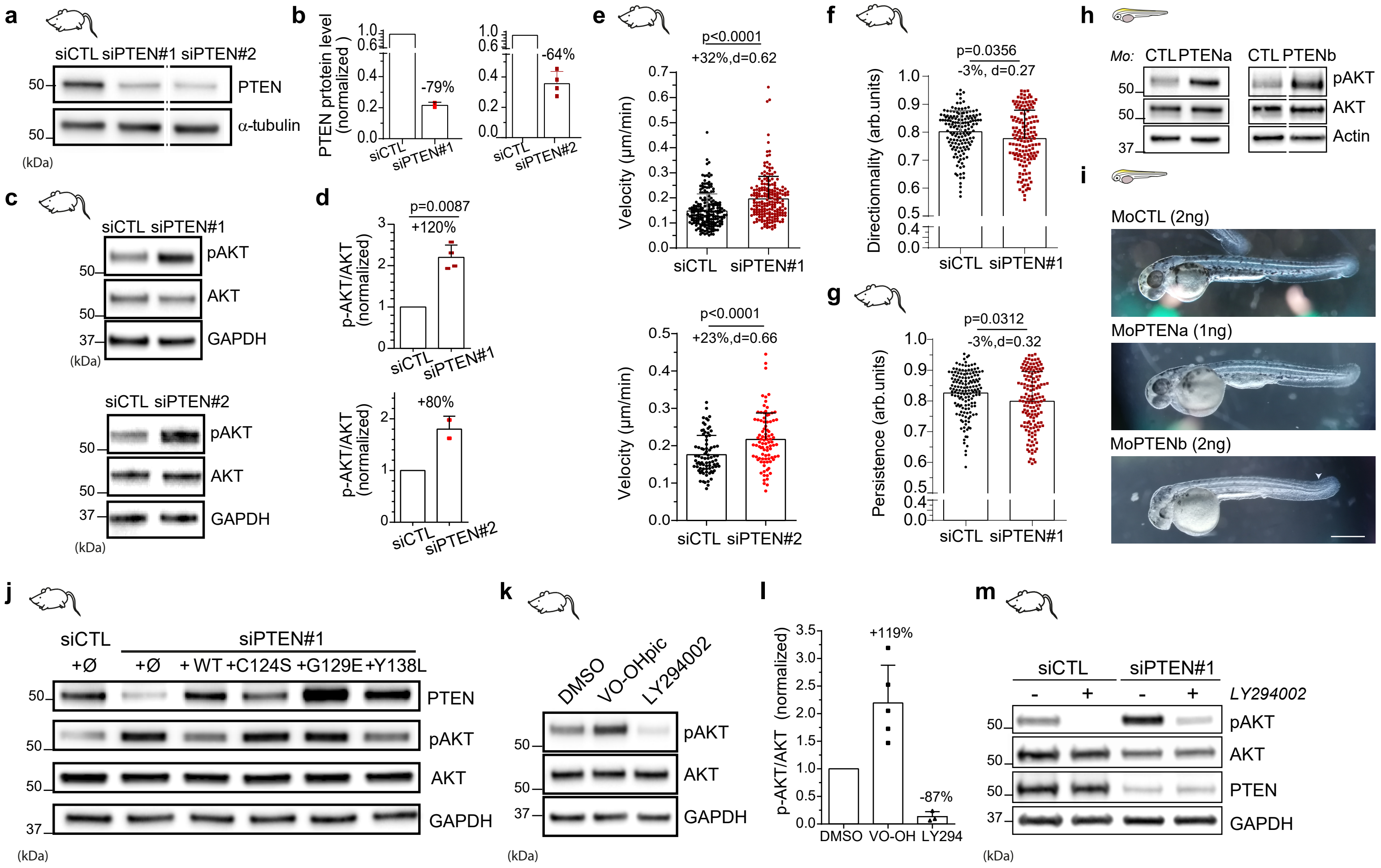
a
siCTL siPTEN#1 siPTEN#2
PTEN
α-tubulin
(kDa)
b
PTEN prtotein level (normalized)
-79%
-64%
siCTL siPTEN#1
siCTL siPTEN#2
c
siCTL siPTEN#1
pAKT
AKT
GAPDH
siCTL siPTEN#2
d
p=0.0087
+120%
p-AKT/AKT (normalized)
+80%
e
p<0.0001
+32%,d=0.62
Velocity (µm/min)
siCTL siPTEN#1
p<0.0001
+23%,d=0.66
siCTL siPTEN#2
f
p=0.0356
-3%, d=0.27
Directionnality (arb.units)
g
p=0.0312
-3%,d=0.32
Persistence (arb.units)
h
Mo: CTL PTENa CTL PTENb
pAKT
AKT
Actin
i
MoCTL (2ng)
MoPTENa (1ng)
MoPTENb (2ng)
j
siCTL siPTEN#1
+Ø +Ø + WT +C124S +G129E +Y138L
PTEN
pAKT
AKT
GAPDH
k
DMSO VO-OHpic LY294002
pAKT
AKT
GAPDH
l
+119%
-87%
p-AKT/AKT (normalized)
DMSO VO-OH LY294
m
siCTL siPTEN#1
LY294002
pAKT
AKT
PTEN
GAPDH

Supplementary Figure 2.

a. Western blot analysis of PTEN, N-cadherin, p120-catenin, αE-catenin, β-catenin and α-tubulin in siCTL and siPTEN#1/#2 astrocytes lysates. **b**. Immunofluorescence images of N-cadherin (gray) and nucleus (DAPI, red) in migrating siCTL and siPTEN#1 astrocytes, highlighting increased distance between leading edge and first lateral cell-cell junction (blue line) in siPTEN#1 leader cells compared to siCTL. Scale bar: 10µm. **c.** Distances between leading edge and first cell-cell junction in siCTL and siPTEN#1 astrocytes (n=145 cells from 3 biological replicates, two-tailed paired t-test). The increased distance in siPTEN#1 suggest alteration of N-cadherin polarised recycling. Error bars represent SD. Full scan images of the immunoblots and source data are provided as a Source Data file

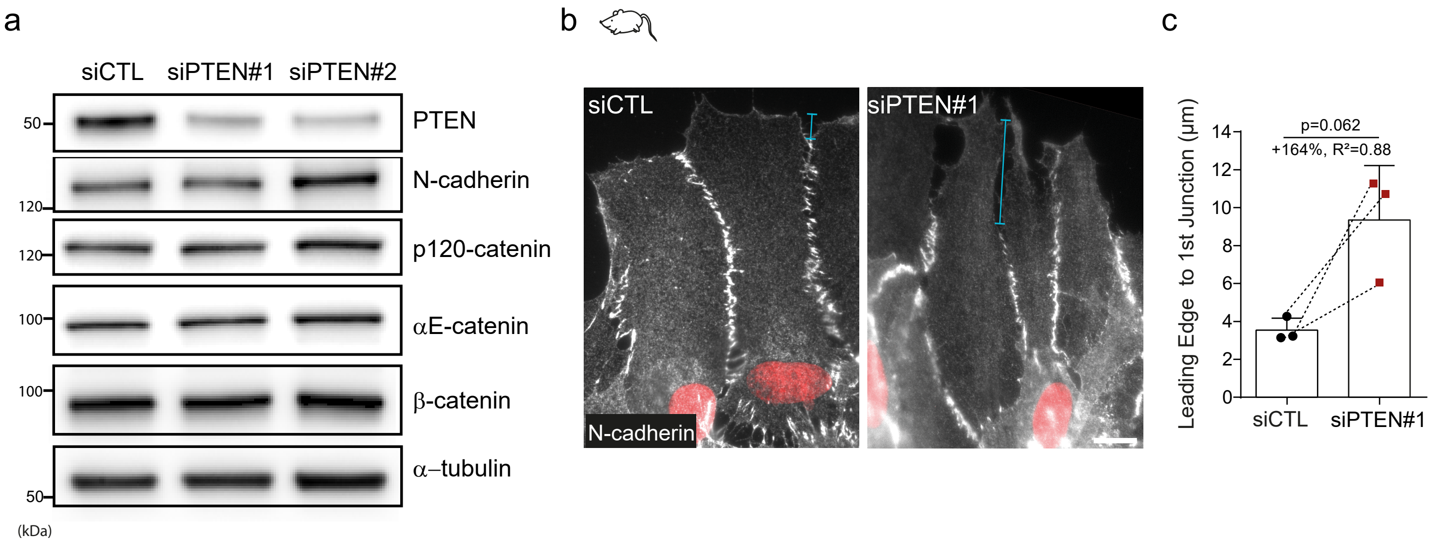
a
siCTL
siPTEN#1
siPTEN#2
PTEN
N-cadherin
p120-catenin
αE-catenin
β-catenin
α–tubulin
50
120
120
100
100
50
(kDa)
b
siCTL
siPTEN#1
N-cadherin
c
Leading Edge to 1st Junction (µm)
p=0.062
+164%, R²=0.88
0
2
4
6
8
10
12
14
siCTL
siPTEN#1

Supplementary Figure 3.

a. Protein phosphorylation Screen Assay (human Phospho-kinase array kit) reveals PTEN targets affected by PTEN depletion (siPTEN#1) but not by PTEN lipid phosphatase inhibition (VO-OH). 41 biotinylated phospho-antibodies spotted in duplicate on nitrocellulose membranes are visualized using chemiluminescent detection reagents. Red boxes highlight phospho-AMPKα1/2 signals, which are zoomed in and transformed into multicolour range pixel intensities. Blue asterisks highlight pAKT as a positive control of the assay. **b.** Table representing FAK, AMPKα and AKT phosphorylation ratios siPTEN#1/siCTL and VO-OH/DMSO. Increased AMPKα phosphorylation is only observed when PTEN is depleted, not when only its lipid phosphatase activity is inhibited. This is the opposite to AKT phosphorylation and similar to FAK phosphorylation, a known PTEN protein phosphatase target. **c.** Western blot analysis of pAMPK, AMPK and GAPDH in siCTL and siPTEN#2. Bands are from same blot but not originally next to each other. **d**. Normalized pAMPK/AMPK ratio showing increased AMPK phosphorylation in siPTEN#2 (n=3 biological replicates, two-tailed paired t-test). **e.** Western blot analysis of pACC, ACC and GAPDH in siCTL and siPTEN#2. Bands in **c.** and **e.** are from same blot but not originally next to each other **f.** Normalized pACC/ACC ratio showing increased AMPK activity in siPTEN#2 (n=3, two-tailed paired t-test). **g**. Representative western blot analysis of co-immunoprecipitation assays in HEK-293T cells transfected with LKB1-GFP and PTEN-HA showing interaction between LKB1 and PTEN (n=2 biological replicates). Error bars represent SD. Full scan images of the immunoblots and source data are provided as a Source Data file.

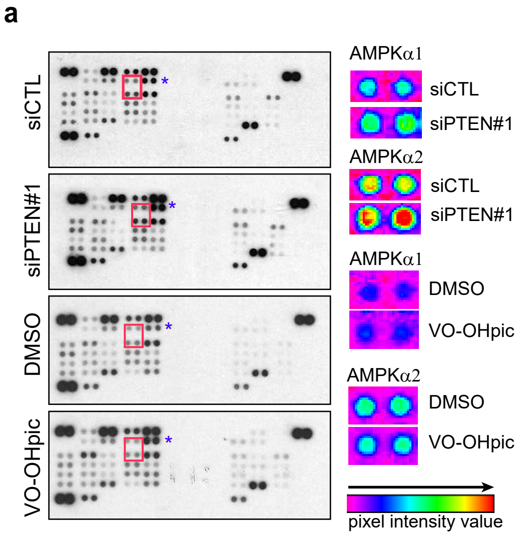

b

	siPTEN#1 / siCTL	VO-OHpic / DMSO
FAK (Y397)	+10%	-15%
AMPKα1/2 (T172)	+17%	-16%
*AKT 1/2/3 (S473)	+37%	+484%

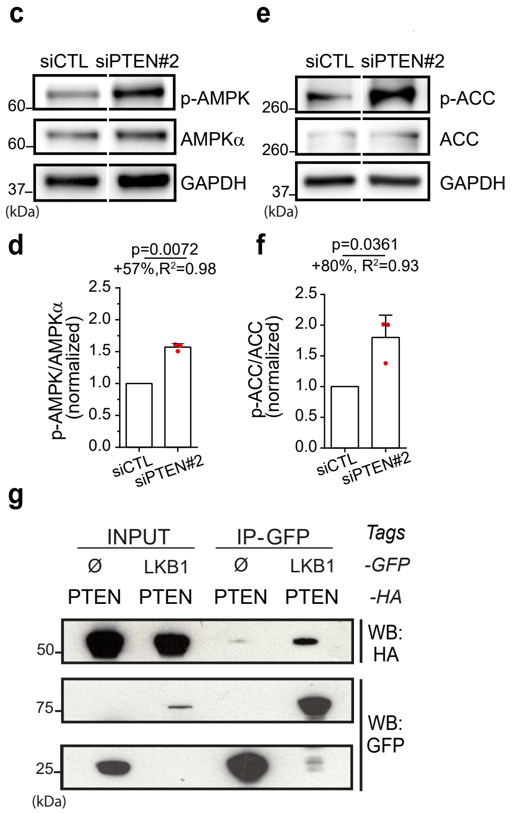

Supplementary Figure 4.

a. Immunofluorescence images of VASP (green), Paxillin (red), N-cadherin (magenta) and nucleus (blue) in migrating siCTL and siPTEN#1 astrocytes. Boxed region highlight recruitment of VASP both at cell-cell junctions (white pixels in the merge channel) and at focal adhesions (yellow pixels in the merge channel) during cell migration in siCTL cells. Note that in siPTEN#1 cells, VASP localises with Paxillin at focal adhesions but not with N-cadherin at cell-cell junctions (yellow asterisks). **b.** Immunofluorescence images of VASP (green), Paxillin (red), F-actin (blue) and nucleus (magenta) in migrating siCTL and siPTEN#1 astrocytes. Boxed region highlight two focal adhesions at the cell front where VASP colocalizes with paxillin and F-actin. No change is observed in siPTEN#1 cells. **c**. Immunofluorescence images of VASP (green), N-cadherin (red), F-actin (blue or gray) in migrating control astrocytes. Yellow dashed lines delineate a zone where interjunctional transverse actin arcs (ITA) are visible and where VASP signal is high at cell-cell junctions. Yellow arrowheads in zoomed in rectangle emphasizes the correlation between VASP and N-cadherin colocalisation and ITA anchoring. Yellow asterisks highlight a zone where no ITA is visible and where VASP signal is absent from cell-cell junctions. **d.** Immunofluorescence images of VASP (green), N-cadherin (red) and F-actin (gray) in siCTL and siPTEN#1 migrating astrocytes. Note the absence of VASP at cell-cell junctions (yellow asterisks) associated with absence of ITA in siPTEN#1 cells. Scale bars: 10µm. Representative images of n=3 biological replicates.

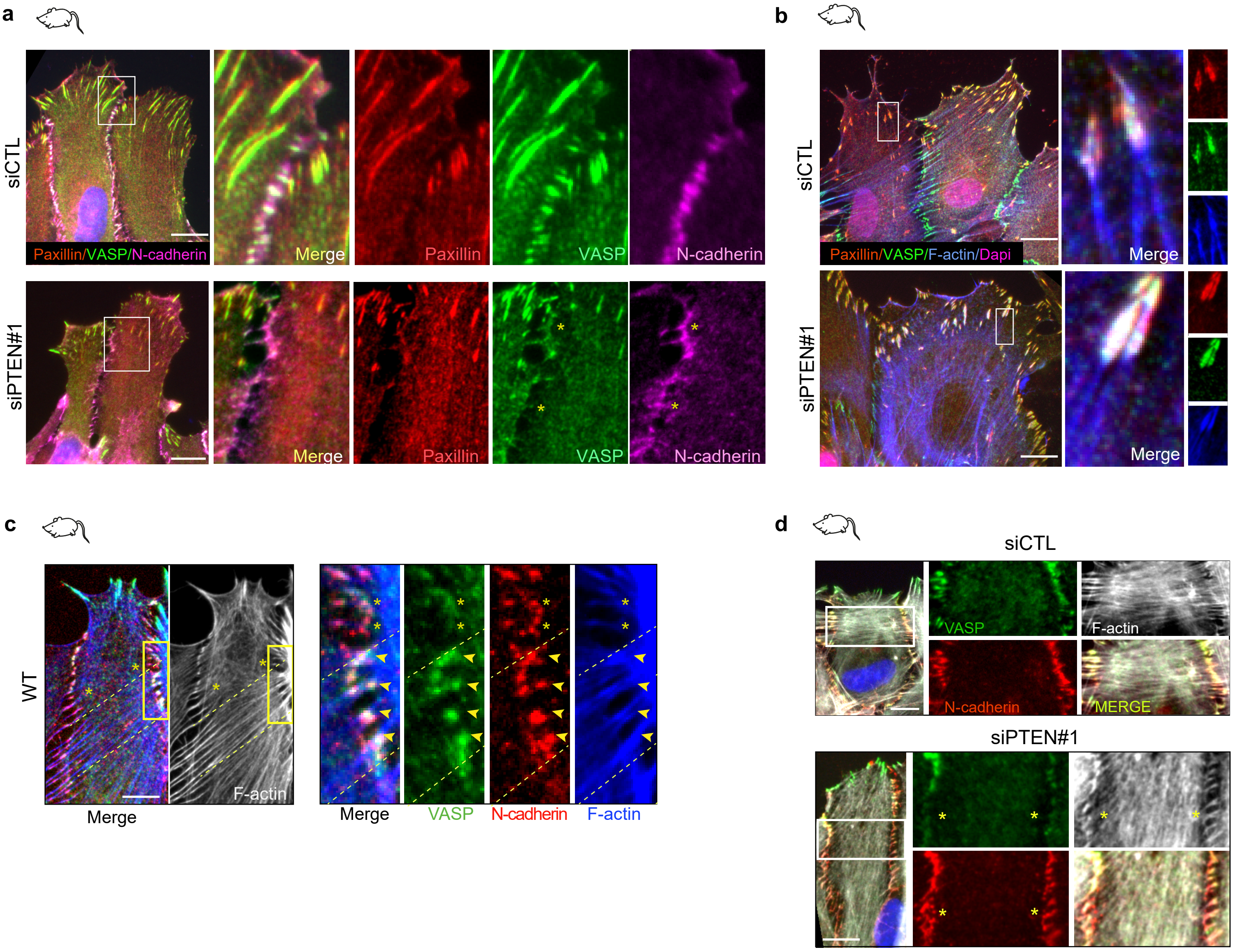

Peglion and Capuana,et al

Supplementary Figure 5.

a. Normalized AMPKα level in siCTL and siAMPKα astrocytes (n=2, biological replicates). **b**. Representative western blot analysis of AMPKα, AMPKβ, pAMPKα, PTEN and α-tubulin in siCTL, siPTEN#2, siAMPKβ, and siPTEN#2 + siAMPKβ (n=2 biological replicates). Note that AMPKβ depletion leads to a decrease in active AMPK (total p-AMPK level). **c.** Mean cell velocity of siPTEN#2 (n=70 cells) and siPTEN#2+siAMPKβ (n=76 cells) migrating astrocytes in wound healing assay. Data are derived from 2 biological replicates**.** Depletion of AMPKβ in siPTEN#2 cells decreases significantly cell migration speed of siPTEN#2 cells. **d.** Representative western blot analysis of PTEN and α-tubulin levels in human Neural Stem Cells (hNSC), rat astrocytes (Astros), U3013 and N13-1520 primary GBM cells showing complete absence of PTEN protein in GBM cells (n=2 biological replicates). **e.** Normalized AMPKβ1 level in shCTL and shAMPKβ1 U3013 GBM cells (n=2 biological replicates).

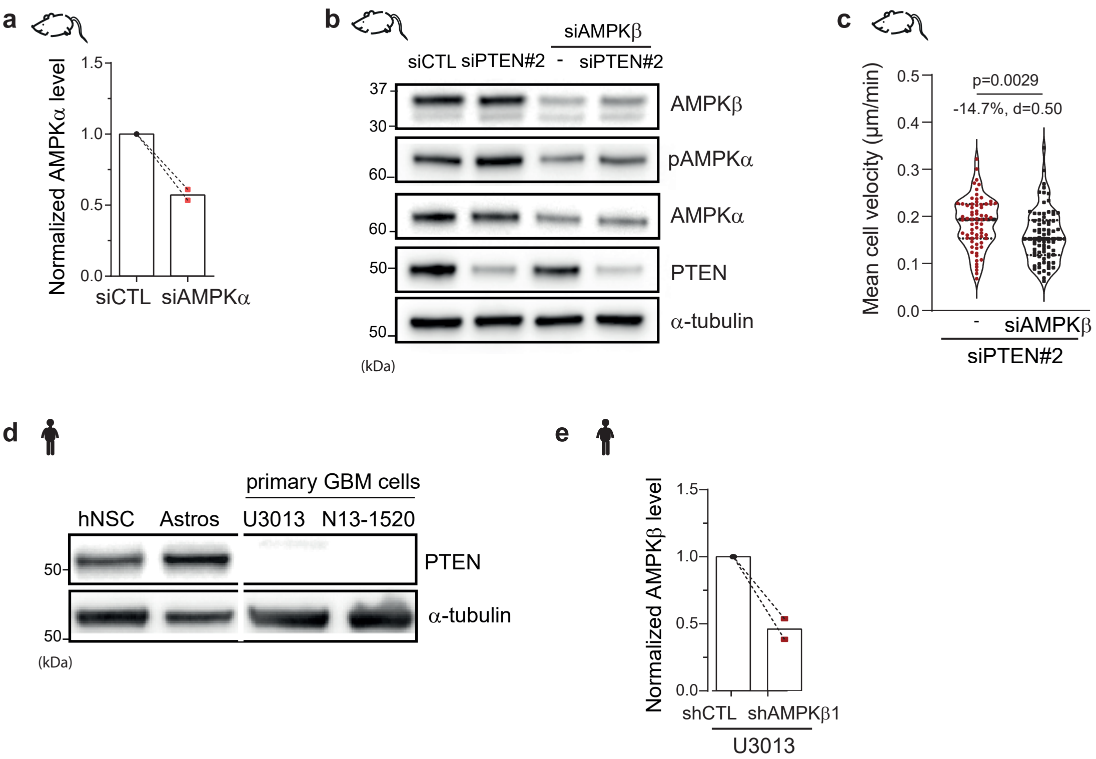
a
Normalized AMPKα level
1.5
1.0
0.5
0.0
siCTL
siAMPKα
b
siAMPKβ
siCTL
siPTEN#2
-
siPTEN#2
37
30
60
60
50
50
AMPKβ
pAMPKα
AMPKα
PTEN
α-tubulin
(kDa)
c
Mean cell velocity (µm/min)
0.5
0.4
0.3
0.2
0.1
0.0
p=0.0029
-14.7%, d=0.50
-
siAMPKβ
siPTEN#2
d
primary GBM cells
hNSC
Astros
U3013
N13-1520
50
50
PTEN
α-tubulin
(kDa)
e
Normalized AMPKβ level
1.5
1.0
0.5
0.0
shCTL
shAMPKβ1
U3013
