# Supplementary material for: PTEN inhibits AMPK to control collective migration
Source: Nat Commun. 2022 Aug 11;13:4528. doi: 10.1038/s41467-022-31842-y (PMC9372137; doi:10.1038/s41467-022-31842-y)
Supplement: Supplementary file 2 — Description of Additional Supplementary Files [file 41467_2022_31842_MOESM2_ESM.pdf]

Description of Additional Supplementary Files

File Name: Supplementary Movie 1
Description: **PTEN depletion increases collective glial cell migration velocity.** siPTEN astrocytes close artificial 2D wound faster than siCTL cells. Total time: 22hours, 1 image every 15min. Scale bar: 100µM.

File Name: Supplementary Movie 2
Description: **AMPK depletion decreases collective cell migration velocity of siPTEN cells.** siCTL, siPTEN#1, siAMPKα1 and siPTEN#1+siAMKPKα1 glial cells closing artificial 2D wounds. Total time: 21hours, 1 image every hour. Scale bar: 100µM.

File Name: Supplementary Movie 3
Description: **AMPK inhibition slows down PTEN-negative primary glioblastoma cell invasion**. U3013 cells are embedded in Matrigel as spheroids and treated with DMSO (left) and Compound C (CC, right). Note that CC treatment prevents the radial invasion of Matrigel observed in DMSO-treated GBM cells. Total time: 24.5hours, 1 image every 15min. Scale bar: 100µM.
