# Supplementary material for: PTEN inhibits AMPK to control collective migration
Source: Nat Commun. 2022 Aug 11;13:4528. doi: 10.1038/s41467-022-31842-y (PMC9372137; doi:10.1038/s41467-022-31842-y)
Supplement: Supplementary file 6 — Reporting Summary [file 41467_2022_31842_MOESM6_ESM.pdf]

nature portfolio

Corresponding author(s): ETIENNE-MANNEVILLE Sandrine

Last updated by author(s): Jun 1, 2022

Reporting Summary

Nature Portfolio wishes to improve the reproducibility of the work that we publish. This form provides structure for consistency and transparency in reporting. For further information on Nature Portfolio policies, see our Editorial Policies and the Editorial Policy Checklist.

Statistics

For all statistical analyses, confirm that the following items are present in the figure legend, table legend, main text, or Methods section.

n/a	Confirmed	
☐	☒	The exact sample size (*n*) for each experimental group/condition, given as a discrete number and unit of measurement
☐	☒	A statement on whether measurements were taken from distinct samples or whether the same sample was measured repeatedly
☐	☒	The statistical test(s) used AND whether they are one- or two-sided<br>*Only common tests should be described solely by name; describe more complex techniques in the Methods section.*
☒	☐	A description of all covariates tested
☒	☐	A description of any assumptions or corrections, such as tests of normality and adjustment for multiple comparisons
☐	☒	A full description of the statistical parameters including central tendency (e.g. means) or other basic estimates (e.g. regression coefficient) AND variation (e.g. standard deviation) or associated estimates of uncertainty (e.g. confidence intervals)
☐	☒	For null hypothesis testing, the test statistic (e.g. *F*, *t*, *r*) with confidence intervals, effect sizes, degrees of freedom and *P* value noted<br>*Give P values as exact values whenever suitable.*
☒	☐	For Bayesian analysis, information on the choice of priors and Markov chain Monte Carlo settings
☒	☐	For hierarchical and complex designs, identification of the appropriate level for tests and full reporting of outcomes
☒	☐	Estimates of effect sizes (e.g. Cohen's *d*, Pearson's *r*), indicating how they were calculated

Our web collection on statistics for biologists contains articles on many of the points above.

Software and code

Policy information about availability of computer code

Data collection	Nikon microscopes coupled with Metamorph®,UltraViewVox Perkin-Elmer microscope coupled with Volocity software and Leica microscopes coupled with Leica Application Software LAS AF 2.4 (Leica Application Suite) were used for image acquisition.
Data analysis	ImageJ version 1.52n was used for all image analysis and processing.<br>Graphpad Prism 7 was used for data representation and statistical analyses.

For manuscripts utilizing custom algorithms or software that are central to the research but not yet described in published literature, software must be made available to editors and reviewers. We strongly encourage code deposition in a community repository (e.g. GitHub). See the Nature Portfolio guidelines for submitting code & software for further information.

Data

Policy information about availability of data

All manuscripts must include a data availability statement. This statement should provide the following information, where applicable:

- Accession codes, unique identifiers, or web links for publicly available datasets
- A description of any restrictions on data availability
- For clinical datasets or third party data, please ensure that the statement adheres to our policy

The data supporting the findings of this study are available within the manuscript . Non-cropped and unprocessed westernblots and all the data used to generate the graphs of the main and supplementary figures can be found in the corresponding source data files. All the raw data generated during this study are available from the corresponding author on reasonable request.

Field-specific reporting

Please select the one below that is the best fit for your research. If you are not sure, read the appropriate sections before making your selection.

☒ Life sciences ☐ Behavioural & social sciences ☐ Ecological, evolutionary & environmental sciences

For a reference copy of the document with all sections, see nature.com/documents/nr-reporting-summary-flat.pdf

Life sciences study design

All studies must disclose on these points even when the disclosure is negative.

Sample size	The sample size was limited by experimental constraints. The authors however estimated that the size was sufficiently high to represent faithfully the total population by considering the dispersion of values and their density around the most frequent value.
Data exclusions	No data were excluded from the analyses.
Replication	All experiments were reproduced independently at least 3 times (unless otherwise stated).
Randomization	All biochemical samples were analyzed. For cell analysis, cells were randomly chosen and imaged without any bias. Primary cells were produced from different independent litters. In vivo studies were repeated on embryos obtained on different days.
Blinding	Data acquisition and analysis were non blinded. All acquired data were analysed (no data exclusion). Automatic quantitative measurements by the relevant softwares and devices did not require blinding.

Reporting for specific materials, systems and methods

We require information from authors about some types of materials, experimental systems and methods used in many studies. Here, indicate whether each material, system or method listed is relevant to your study. If you are not sure if a list item applies to your research, read the appropriate section before selecting a response.

Materials & experimental systems

n/a	Involved in the study	
☐	☒	Antibodies
☐	☒	Eukaryotic cell lines
☒	☐	Palaeontology and archaeology
☐	☒	Animals and other organisms
☒	☐	Human research participants
☒	☐	Clinical data
☒	☐	Dual use research of concern

Methods

n/a	Involved in the study	
☒	☐	ChIP-seq
☒	☐	Flow cytometry
☒	☐	MRI-based neuroimaging

Antibodies

Antibodies used	The following primary antibodies were used: anti–α-tubulin (1/500, MCA77G YL1/2, rat monoclonal; Bio-Rad), anti-GAPDH (1/2000, mouse, Chemicon International, MAB374), anti-Paxillin (1/200, mouse, BD transduction, #610051), anti–N-cadherin (1/1000, ab12221, rabbit polyclonal, lot GR139340-26, Abcam and sc-31030, 1/200 for immunofluorescence, goat polyclonal, clone K-20, lot B2014, Santa-Cruz), anti-αE-catenin (1/1000, Rabbit, Cell Signaling 3236S), anti-βcatenin (1/1000, Mouse, BD,#610154, lot:3137536), anti-p120ctn (1/1000, Mouse, BD, #610134, lot:71996), anti-P-Akt (1/1000e, Rabbit, Cell Signaling 4060S), anti-Akt (1/1000, rabbit, Cell Signaling 4685S), anti-AMPKα (1/1000, Rabbit, Cell Signaling 2532S), anti-AMPKβ (1/1000, Rabbit, Cell Signaling 4150S), p-AMPKα/T172 (1/1000, Rabbit, Cell Signaling 2585S), ACC (1/1000e, Rabbit, Cell Signaling, 3662S), p-ACC (1/1000, Rabbit, Cell Signaling 3661S), LKB1 (1/1000, Rabbit, Cell Signaling, 3047),VASP (1/1000 for immunoblot and 1/200 for immunofluorescence, Rabbit, Cell Signaling 3132), P-VASP/T278 (1/1000, Rabbit, Sigma Aldrich, SAB4200521), GFP-HRP (1/5000, Abcam, Ab6663). Phalloidin-iFluor647 reagent (1/400, Abcam Ab176759) was used to label F-actin filaments. Secondary antibodies were Alexa Fluor 488 donkey anti–rabbit (711-545-152), Rhodamine (TRITC) donkey anti–rabbit (711-025-152), Rhodamine (TRITC) donkey anti-mouse (715-025-151), Alexa Fluor 647 donkey anti-rabbit (711-695-152), Alexa Fluor 647 donkey anti–goat (705-605-147), and Alexa Fluor 488 donkey anti–rat (712-545-153); from Jackson ImmunoResearch, all at a dilution of 1/10000.
Validation	Antibodies were validated by the manufacturer. We verified that the antibodies showed proteins of the correct size by westernblotting. Potential anibody crossreaction, and antibody validity with rat proteins was evaluated by using rat and human cells in parallel and by testing samples obtained from cells in which the protein of interest had been depleted or overexpressed in a tagged version.

Eukaryotic cell lines

Policy information about cell lines

Cell line source(s)	HEK-293T (ATCC #CRL-11268TM); U-87 MG (ECACC 89081402); U-373MG (Uppsala, ECACC 08061901); U3013 (Uppsala, HGCC, Xie et al., 2015), N13-1520 (Gliotex, ICM, Paris, France. Rosenberg et al, 2016)
Authentication	The cell line was not authenticated after purchase
Mycoplasma contamination	Cell lines regularly tested negative for Mycoplasma contamination.
Commonly misidentified lines (See ICLAC register)	No commonly misidentified cell lines

Animals and other organisms

Policy information about studies involving animals; ARRIVE guidelines recommended for reporting animal research

Laboratory animals	Primary astrocytes were obtained from E17 OFA rat embryos (Charles River, Wilmington, MA, USA). Zebrafish (Danio Rerio) of the AB background (Wt, from the Zebrafish International Resource Center) IRC, Eugene, OR, USA. Only rat embryos and fish larvae are used, the sex is unknown. Zebrafish larvae were injected at 3days post fertilization (as indicated in the manuscript)
Wild animals	No wild animals were used in this study
Field-collected samples	This study did not involve any field collected samples
Ethics oversight	Use of these animals is in compliance with ethical regulations and has been approved from the Prefecture de Police and Direction departementale des services veterinaires de Paris. All protocols with the zebrafish animals were approved by the Ethical Committee for Animal Experimentation of Institut Pasteur – CEEA 89 and the French Ministry of Research and Education (permit #01265.03).

Note that full information on the approval of the study protocol must also be provided in the manuscript.
